# Thirty-Day Readmission Rates after Takotsubo Syndrome with or without Malignancy: A Nationwide Readmissions Database Analysis

**DOI:** 10.3390/jcm10163701

**Published:** 2021-08-20

**Authors:** Sun-Joo Jang, Ilhwan Yeo, Chanel Jonas, Parag Goyal, Jim W. Cheung, Dmitriy N. Feldman, S. Andrew McCullough, Udhay Krishnan, David L. Narotsky, Harsimran S. Singh, Robert M. Minutello, Geoffrey Bergman, S. Chiu Wong, Luke K. Kim

**Affiliations:** 1 Weill Cornell Cardiovascular Outcomes Research Group (CORG), Department of Medicine, Division of Cardiology, Weill Cornell Medicine, New York Presbyterian Hospital, New York, NY 10065, USA; ckj9002@nyp.org (C.J.); pag9051@med.cornell.edu (P.G.); jac9029@med.cornell.edu (J.W.C.); dnf9001@med.cornell.edu (D.N.F.); stm9150@med.cornell.edu (S.A.M.); udk9001@med.cornell.edu (U.K.); david.narotsky@gmail.com (D.L.N.); has9028@med.cornell.edu (H.S.S.); rmm2002@med.cornell.edu (R.M.M.); geb2003@med.cornell.edu (G.B.); scwong@med.cornell.edu (S.C.W.); luk9003@med.cornell.edu (L.K.K.); 2 Department of Internal Medicine, Yale New Haven Health, Bridgeport Hospital, Bridgeport, CT 06610, USA; 3 Division of Cardiology, New York Presbyterian Queens Hospital, New York, NY 11355, USA; iy2159@caa.columbia.edu

**Keywords:** Takotsubo syndrome, malignancy, readmission, cardio-oncology

## Abstract

The association between malignancy and readmission after Takotsubo syndrome (TTS) hospitalization has not been fully described. We sought to examine the rates, cause, and cost of 30-day readmissions of TTS, with or without malignancy, by utilizing Nationwide Readmissions Databases from 2010 to 2014. We identified 61,588 index hospitalizations for TTS. TTS patients with malignancy tended to be older (70.6 ± 0.2 vs. 66.1 ± 0.1, *p* < 0.001), and the overall burden of comorbidities was higher than in those without malignancy. TTS patients with malignancy had significantly higher 30-day readmission rates than those without malignancy (15.9% vs. 11.0%; odds ratio (OR), 1.35; 95% confidence interval (CI), 1.18–1.56). Non-cardiac causes were the most common causes of readmission for TTS patients with malignancy versus without malignancy (75.5% vs. 68.1%, *p* < 0.001). The 30-day readmission rate due to recurrent TTS was very low in both groups (0.4% and 0.5%; *p* = 0.47). The total costs were higher by 25% (*p* < 0.001) in TTS patients with vs. without malignancy. In summary, among patients hospitalized with TTS, the presence of malignancy was associated with increased risk of 30-day readmission and increased costs. These findings highlight the importance of optimized management for TTS patients with malignancy.

## 1. Introduction

The Takotsubo syndrome (TTS), also known as stress-induced cardiomyopathy, can mimic acute coronary syndrome (ACS) and is an increasingly recognized cause of heart failure [1,2]. There are multiple theories regarding the pathophysiology of TTS, though the exact mechanisms remain uncertain [3]. The association between malignancy/chemotherapy and TTS has been reported in multiple studies [4,5,6]. Cancer patients who undergo systemic chemotherapy and/or radiation therapy often develop endothelial dysfunction in their epicardial and microvascular coronary vasculatures, which may play an important role in the high frequency of TTS observed in this cohort.

A recent study demonstrated a high prevalence of malignancy among TTS patients with an increased long-term mortality in TTS patients with concomitant malignancy [4,5]. Although TTS has been shown to be associated with frequent (12%) 30-day readmission, the clinical impact of readmission on TTS patients with malignancy and its associated economic burden on the US healthcare system is less evident [6,7]. Using the Nationwide Readmission Database (NRD), we sought to delineate the incidence, etiology, clinical impact, and costs of 30-day readmission in TTS patients with versus without malignancy.

## 2. Methods

### 2.1. Data Source

Data were obtained from the Agency for Healthcare Research and Quality, which administers the Healthcare Cost and Utilization Project (HCUP) [8,9]. We used the NRD from 2010 to 2014. The NRD is a large, administrative database constructed using discharge data from HCUP State Inpatient Databases. It has verified patient linkage numbers used to track the patients across hospitals within a state during a given year. The NRD is designed to support national readmission analyses and is a publicly available and nationally representative healthcare database. From 2010 to 2014, the NRD contained deidentified information for a total of 70,501,787 index hospitalizations from 1715–2048 hospitals in 18–20 states, representing a national estimate of 181,545,077 discharges. Each patient record in the NRD contains information on the patient’s diagnoses and procedures performed during the hospitalization, based on International Classification of Diseases, Ninth Revision-Clinical Modification (ICD-9-CM) codes and Clinical Classification Software (CCS) codes that groups multiple ICD-9-CM codes for facilitated statistical analyses. We identified our study population, comorbidities, causes of readmissions and in-hospital outcomes using a combination of ICD-9-CM codes and CCS codes. Institutional Review Board approval and informed consent were not required for the current study because all of the data collection derived from a publicly open and deidentified administrative database.

### 2.2. Study Population and Variables

From 2010 to 2014, all index hospitalizations for TTS were selected using ICD-9-CM code 429.83. To ensure that cases were TTS and not ACS, only those who underwent diagnostic coronary angiography (CCS 47) and no revascularization (Procedural CCS 44, 45 and ICD-9-CM procedure codes 36.06 and 36.07) were included in the analysis. Malignancy was identified by CCS code 11–45 [10]. Patients were categorized into 2 groups based on the presence or absence of malignancy.

Patient- and hospital-level variables were included as baseline characteristics. Patients with a concomitant diagnosis of cardiogenic shock and cardiac arrest were identified using ICM-9-CM codes 785.51 and 427.5, respectively. Concurrent use of intra-aortic balloon pump and percutaneous left ventricular assist devices were identified with ICD-9-CM procedure codes 37.61 and 37.68, respectively. Since NRD prohibits linking patients across years, patients whose index hospitalization was in December were excluded in order to allow for completeness of data on 30 days of follow-up after discharge, similarly to other prior studies examining the NRD [11,12,13].

### 2.3. Study Outcomes

The primary outcome of interest was 30-day readmission rates after index hospitalization for TTS. For 30-day readmissions, only the first readmission within 30 days of the discharge was included, and transfer to another hospital was not counted as a readmission. The primary cause of 30-day readmission was identified based on CCS code in the first diagnosis field of each readmission record and dichotomized into non-cardiac and cardiac causes [9]. Non-cardiac causes included respiratory, infectious, gastrointestinal, neuropsychiatric/substance, stroke/transient ischemic attack, endocrine/metabolic, genitourinary, hematologic/oncologic, peripheral vascular disease, trauma, complication of medical procedure, and other non-cardiac causes. Cardiac causes included angina and chronic ischemic heart disease, heart failure, acute myocardial infarction, nonspecific chest pain, arrhythmia, and other cardiac causes. Furthermore, 30-day mortality rates, along with breakdown of mortality rates during index hospitalization and during readmission, were identified. Finally, total cumulative hospital charges and costs for index hospitalization and readmissions were examined according to the presence of malignancy.

### 2.4. Statistical Analyses

All statistical analyses were performed using SAS software, version 9.4 (SAS Institute, Cary, NC, USA), and R statistical software, version 3.5.1 (www.R-project.org (accessed on 1 May 2021)), with its package “survey”. Discharge weight and stratum provided by NRD were used for all analyses and thus all of the reported numbers are weighted national estimates [14]. Domain analysis was used for accurate variance calculations for subgroup analyses [15]. All analyses accounted for NRD sampling design by including hospital-year fixed effects based on hospital identification number [8]. We compared baseline patient- and hospital-level characteristics with TTS, stratified by the presence of malignancy. Categorical variables are presented as frequencies and analyzed by the Rao-Scott chi-square test. Continuous variables are shown as mean or median and are tested by either the Mann-Whitney-Wilcoxon test or a survey-specific linear regression test. To evaluate the predictive value of malignancy and other covariates for primary and secondary outcomes, survey-specific univariate and multivariable generalized linear models were applied. Variables with *p* < 0.1 were included as initial covariates. Final parsimonious models were created by manual removal of each covariate, based on Akaike information criterion, while ensuring each removal did not result in >10% change in the measure of association for the primary predictor variable. Adjusted risks are presented in odds ratio (OR), together with 95% confidence interval (CI) and *p* value. The estimated cost for each hospitalization was calculated by the validated method of using cost-to-charge ratio provided by HCUP. The total cost was defined as the summation of the cost of readmission and the cost of the index admission. We examined the predictors of total cost by survey-specific multivariate linear regression test for log-transformed costs. All tests were two-sided with *p* value < 0.05 considered statistically significant.

## 3. Results

### 3.1. Baseline Characteristics

A total of 61,583 TTS admissions were included in the analysis, with 7542 patients (12.2%) with malignancy. The baseline patient-level and hospital-level characteristics, according to the presence of malignancy, are presented in Table 1. TTS patients with malignancy were more likely to be female and have a smoking history, known coronary artery disease, congestive heart failure, chronic pulmonary disease, anemia, atrial fibrillation, coagulopathy, fluid/electrolyte disorders, pulmonary circulatory disease and valvular heart disease. TTS with concomitant cardiogenic shock was observed more frequently among those with malignancy. In addition, TTS patients with malignancy were admitted more frequently to teaching hospitals and discharged to facilities more often (such as skilled nursing facility, intermediate care facility, and inpatient rehabilitation facility). There was no significant difference in the prevalence of psychiatric disorders, such as mood disorders, delirium/dementia, personality disorder between TTS patients with malignancy and those without malignancy.

### 3.2. Clinical Outcomes of TTS Patients with or without Malignancy

In-hospital mortality in TTS patients with malignancy was significantly higher by ~2-fold when compared to those without malignancy (4.2% vs. 2.1%, *p* < 0.001) (Table 2). There was more than a 90% increase in the 30-day total mortality rates in the malignancy group, which was mostly driven by mortality during index-hospitalization. Multivariate analysis, after adjusting for clinical and hospital characteristics, demonstrated a 68% increase in the risk of index-hospitalization mortality (OR, 1.68; 95% CI, 1.29–2.17; *p* < 0.01) and a 62% increase in the risk of 30-day total mortality (OR, 1.62; 95% CI, 1.25–2.10; *p* < 0.01) in TTS patients with vs. without malignancy (Table 2, Tables S1 and S2). There was no difference in 30-day readmission mortality between TTS patients with and without malignancy (0.6% vs. 0.4%, *p* = 0.110).

**Figure 1:**
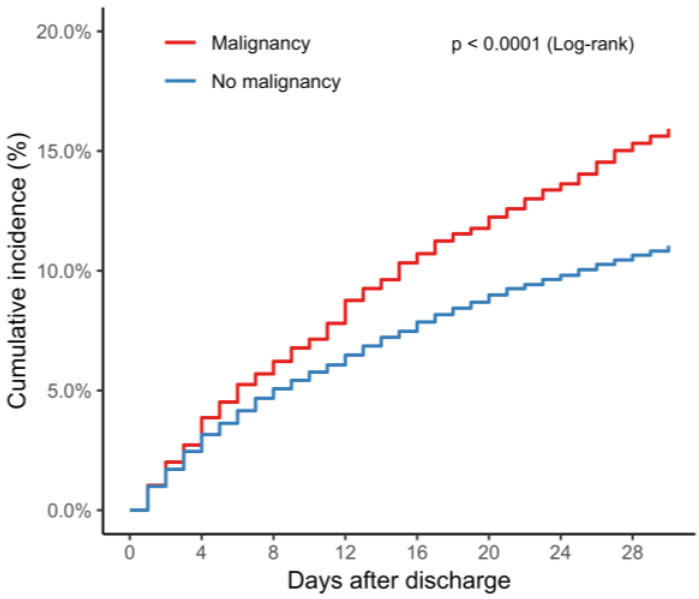
Cumulative rates of 30-day readmissions according to the presence of malignancy. Data show unadjusted 30-day readmission rate in TTS patients with malignancy (red) and in those without malignancy (blue). Abbreviations: TTS, Takotsubo syndrome.

The 30-day readmission rate was significantly higher in TTS patients with malignancy than those without malignancy (15.9% vs. 11.0%, *p* < 0.001) (Figure 1). After adjusting for clinical and hospital characteristics, there was a 33% increase in the risk of 30-day readmission in TTS patients with malignancy (OR, 1.33; 95% CI, 1.15–1.53; *p* < 0.01) (Table 3). Other significant predictors for 30-day readmission included a longer (>5 days) length of stay during index admission, chronic pulmonary disease, chronic kidney disease, anemia, atrial fibrillation, fluid/electrolyte disturbance, diabetes mellitus, low household income, and disposition to facility (Table 3).

### 3.3. Timing and Cause of Readmission

**Figure 2:**
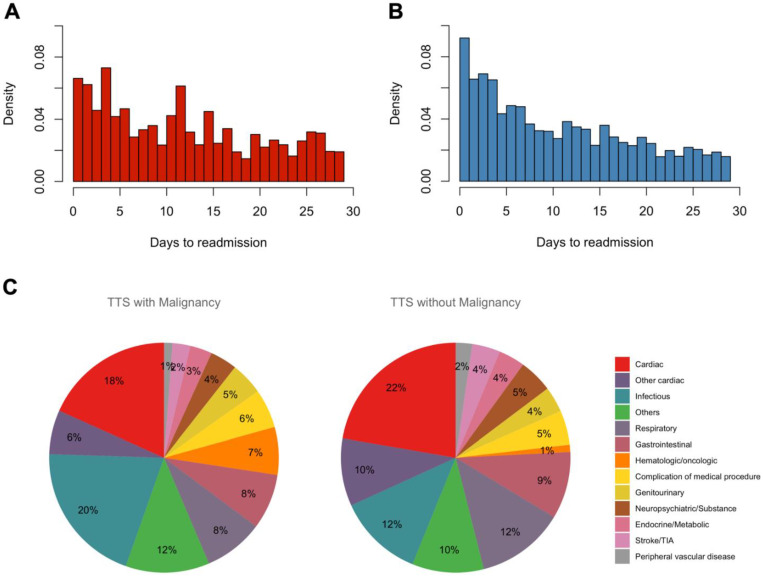
Timing and Causes of 30-day Readmissions for Takotsubo Syndrome with or without malignancy. (**A**) Time to readmission in TTS patients with malignancy. (**B**) Time to readmission in TTS patients without malignancy. (**C**) Histograms representing different causes of 30-day readmissions in TTS patients with malignancy and without malignancy. Abbreviations: TTS, Takotsubo syndrome.

Figure 2 and Figure S1 demonstrate the timing of a 30-day readmission stratified by the presence of malignancy. The median time to readmission was longer in TTS patients with malignancy vs. without malignancy (12 days, interquartile range (IQR) 5–19 days vs. 9 days, IQR 4–17 days, respectively; *p* = 0.027). A sum of 34% of TTS patients with malignancy and 43% without malignancy were readmitted within 7 days of discharge. A sum of 38% of TTS patients with malignancy and 33% without malignancy were readmitted after 14 days of discharge. Non-cardiac causes were more common causes of readmission for TTS patients with malignancy versus without malignancy (75.5% vs. 68.1%, *p* < 0.001) (Figure 2C, Tables S3–S5). Among cardiac causes, heart failure was the most prevalent in both TTS patients with and without malignancy (8.1% vs. 11.1%, *p* = 0.002), followed by arrhythmia (4.9% vs. 3.3%, *p* = 0.006, and angina (1.8% vs. 2.4%, *p* = 0.234). Among non-cardiac causes, infectious (20.1% vs. 12.0%, *p* = <0.001), respiratory (8.4% vs. 12.4%, *p* < 0.001), and gastrointestinal (7.8% vs. 9.5%, *p* = 0.075) causes were most prevalent in both groups. The 30-day readmission rate due to recurrent TTS was similarly low in both groups (0.4% vs. 0.5%, respectively; *p* = 0.47).

### 3.4. Specific Cancer Type and Readmission

**Figure 3:**
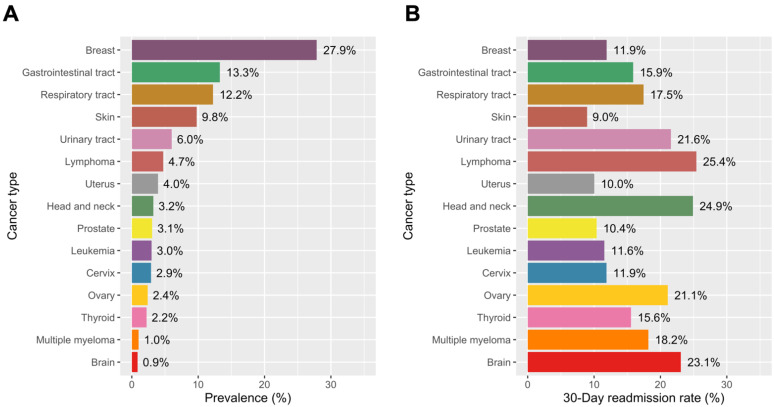
Thirty-day Readmission Rates of Takotsubo Syndrome by Cancer Types. (**A**) Prevalence of each type of cancer in TTS patients with malignancy. (**B**) 30-day readmission rates according to the cancer type in TTS patients with malignancy. Abbreviations: TTS, Takotsubo syndrome.

The prevalence of specific cancer types is shown in Figure 3A. The most frequent type of malignancy was breast cancer (27.9%), followed by gastrointestinal tract cancer (13.3%) and respiratory tract cancer (12.2%). The 30-day readmission rate was the highest in patients with lymphoma (25.4%), head and neck cancer (24.9%), brain cancer (23.1%), urinary tract cancer (21.6%), and ovarian cancer (21.1%) (Figure 3B). TTS patients with skin (9.0%), uterus (10.0%), and prostate (10.4%) cancer had the lowest 30-day readmission rates compared to patients with other cancer.

### 3.5. Total Charges and Costs by the Presence of Malignancy and Predictors of Total Cost

Hospital charges and costs over 30 days after index hospitalization for TTS stratified by the presence of malignancy are shown in Table S6. The median total charge (index hospitalization + readmissions) was $10,201 higher in TTS patients with malignancy ($50,936; IQR, $29,819–$97,989) than in those without malignancy ($40,735; IQR, $26,160–$72,619) (*p* < 0.001). The median total cost (index hospitalization + readmissions) was $2982 higher in TTS patients with malignancy ($14,686; IQR, $9294–$27,337) than in those without malignancy ($11,704; IQR, $8065–$19,630) (*p* < 0.001). After multivariate adjustment, 30-day readmission was independently associated with a 22.3% increase in the 30-day total cost (Table 4). Furthermore, the presence of malignancy was independently associated with a 1.3% increase in the 30-day total cost. Other significant predictors for the increased total costs included length of stay > 5 days, cardiogenic shock, and cardiac arrest.

## 4. Discussion

Using the NRD between 2010 and 2014 to delineate 30-day clinical outcomes in patients hospitalized for TTS with or without malignancy, we identified several key findings. First, there was a high prevalence of malignancy (12.2%) among TTS patients. Second, 30-day readmission rates, all-cause mortality, and total costs (index hospitalization + readmissions) were higher in TTS patients with malignancy compared to those without malignancy. Third, malignancy was independently associated with an increase in 30-day total charges and costs in patients hospitalized for TTS. The implications of these findings underscore the importance of optimal treatment strategy and close follow-up in patients with TTS and malignancy.

The 30-day readmission rate of TTS patients was 11.6%, similar to previous reports [7,16]. Our study demonstrates that malignancy is associated with a significant risk of readmission after index hospitalization for TTS. It is noteworthy that the median time to readmission is longer in TTS patients with malignancy (12 days) than in those without malignancy (9 days). A significant portion (34%) of TTS patients with malignancy were readmitted >14 days after discharge. These findings highlight the need for optimal initial treatment strategy and vigilant attention beyond the initial 1–2 weeks follow-up visit, especially for patients with malignancy. Our study demonstrates that heart failure is the most common cardiac cause of readmission, which is unsurprising given the pathophysiology and clinical manifestation of TTS. Therefore, treatment strategies to optimize heart failure regimen and to minimize readmission after TTS are warranted [17]. Supportive discharge interventions, such as telephone-facilitated post-discharge support program, computer-based education sessions, or nurse-driven protocol-based management program, have shown to be effective in reducing the risk of early readmissions in the heart failure population [18,19,20,21,22]. Furthermore, developing comprehensive programs for heart failure patients with patient-specific interventions based on risk profile and focus on both inpatient and outpatient interventions with cross-site communications have shown to significantly reduce early readmissions [23]. However, efforts in reducing 30-day readmissions after TTS should also consider the fact that the majority of readmissions are due to non-cardiac causes. TTS is a complex disorder with variable clinical manifestations, and malignancy certainly presents an added layer of complexity to the management of TTS. Infectious etiologies were the most frequent cause of readmission among TTS patients with malignancy in our study. Patients with malignancy are at increased risk of infections directly related to their cancer or due to their immunocompromised state from systemic chemotherapy [24]. Interdisciplinary approaches in coordinating outpatient programs focused on reducing readmissions by providing a continuity of care in the inpatient and outpatient setting by both oncologists and cardiologists should be encouraged.

In the present study, the presence of malignancy is not associated with an increased risk in 30-day readmission from recurrent TTS. Reported recurrence rates of TTS are variable, ranging from 1% to 11.4% [25,26,27]. Analysis of the multicenter GEIST (German Italian Stress Cardiomyopathy) Registry data failed to identify any independent predictors of TTS recurrence [28]. The presence of malignancy has been shown to be associated with a higher risk of TTS in numerous studies [29,30,31], and there are numerous reports of TTS associated with chemotherapy or immunotherapy [32,33,34,35,36]. It is plausible that interruption in chemotherapy after initial episode of TTS may play a role in explaining the lack of difference in TTS recurrence rate in those with malignancy. Post-TTS cancer treatment strategy, especially decisions regarding continuation of potentially life-saving chemotherapy, still remains a challenge. Future, longer-term studies are necessary to detect whether TTS patients with malignancy are at risk of developing recurrent TTS.

Our study demonstrated that malignancy was associated with ~70% increase in in-hospital mortality risk during index hospitalization and ~60% increase in overall 30-day mortality risk in patients with TTS. A previous study from National Inpatient Sample 2007 to 2013 showed that solid cancer was associated with 3.4 times increase in in-hospital mortality among TTS patients [37]. Our analysis included all types of cancer (solid or hematologic cancer), which may explain slightly lower in-hospital mortality. Recent data from the International Takotsubo Registry have shown that long-term mortality increased in TTS patients with malignancy while 30-day mortality was not significantly different in TTS patients (*n* = 1604) with or without malignancy (7% vs. 4%, *p* = 0.17) [29]. Our larger administrative data (*n* = 61,583), however, demonstrates that total 30-day mortality rates in TTS patients with malignancy were significantly higher compared to those without malignancy. This result requires a cautious interpretation since the NRD does not include out-of-hospital mortality data. Further studies with a more extensive examination of out-of-hospital mortality are needed to examine this discrepancy.

Cost analysis of our study demonstrates that TTS is responsible for annual cost of ~$148 million for index admission and 30-day readmission in the US (during the years from 2010 to 2014). Readmission within 30-days accounted for 22% increase in the total costs among TTS patients. Furthermore, the presence of malignancy increased the cumulative costs by 1.3% after index hospitalization for TTS. Although 30-day readmission is one of the most significant contributors to an increased overall cost, the presence of malignancy was an independent predictor of increased cost, likely due to longer length of stay and higher frequency of cardiogenic shock. Our findings highlight the impact of TTS in patients with malignancy beyond just short- and long-term mortality. TTS in patients with malignancy pose a significant economic burden on hospital resources and the overall healthcare system, and our study supports the need for increased effort in reducing readmissions in this cohort.

This study has limitations that are largely due to its observational nature. Firstly, the NRD is the administrative data, designed to estimate the national distribution of representative hospital characteristics, which is subject to coding bias or missing variables. Nevertheless, there have been numerous publications which have validated the sampling design of NRD and the utilization of NRD databases for risk-adjusted outcome evaluation [11,16,38]. Secondly, the NRD does not include detailed clinical characteristics, such as coronary anatomy, heart failure class, left ventricular ejection fraction, cardiac enzyme data or medications. Thirdly, our analysis of the 30-day total mortality rate does not include out-of-hospital deaths, which may underestimate the overall incidence of mortality. Fourthly, we used ICD-9-CM codes and CCS codes for defining clinical diagnoses and procedures, which may lead to a misclassification bias. Finally, the result from the overall malignant population may not be generalizable to specific cancer types, stages, or treatment strategies. Nevertheless, the prevalence and 30-day readmission rates according to cancer types were addressed throughout this study.

In summary, malignancy is associated with an increased risk of 30-day readmission, all-cause mortality, and costs in TTS patients. The majority of hospital readmissions in TTS patients were caused by non-cardiac causes. Our findings highlight the importance of tailored and multidisciplinary patient-specific treatment approaches, along with careful coordination of outpatient follow-up care, particularly in TTS patients with malignancy.

## Figures and Tables

**Table 1:** Baseline characteristics of Takotsubo Syndrome Patients with or without Malignancy.

Characteristics				
	All	Malignancy	No Malignancy	*p* Value
Number of admissions	61,583	7542 (12.2) *	54,041 (87.8)	
Patient characteristics				
Age, mean (SE), y	66.7 (0.1)	70.6 (0.2)	66.1 (0.1)	<0.001 †
Age ≥ 70 yrs	27,109 (44.0)	4328 (57.4)	22,781 (42.2)	<0.001 ‡
Female	54,708 (88.8)	6499 (86.2)	48,209 (89.2)	<0.001
Smoking history	21,834 (35.5)	2864 (38.0)	18,970 (35.1)	0.005
Hypertension	40,597 (65.9)	4900 (65.0)	35,697 (66.1)	0.350
Diabetes mellitus	11,701 (19.0)	1405 (18.6)	10,296 (19.1)	0.620
Dyslipidemia	26,830 (43.6)	3102 (41.1)	23,728 (43.9)	0.014
Known coronary artery disease	25,910 (42.1)	3495 (46.3)	22,415 (41.5)	<0.001
Previous myocardial infarction	3833 (6.2)	444 (5.9)	3389 (6.3)	0.463
Previous PCI	2988 (4.9)	341 (4.5)	2647 (4.9)	0.458
Previous CABG	933 (1.5)	91 (1.2)	842 (1.6)	0.177
Family history of coronary artery disease	5249 (8.5)	448 (5.9)	4801 (8.9)	<0.001
Congestive heart failure	18,531 (30.1)	2552 (33.8)	15,979 (29.6)	<0.001
Peripheral vascular disease	4544 (7.4)	608 (8.1)	3936 (7.3)	0.156
Chronic pulmonary disease	17,732 (28.8)	2396 (31.8)	15,336 (28.4)	<0.001
Chronic kidney disease	4862 (7.9)	670 (8.9)	4192 (7.8)	0.075
Liver disease	1238 (2.0)	116 (1.5)	1122 (2.1)	0.070
Anemia	9425 (15.3)	1456 (19.3)	7969 (14.7)	<0.001
Atrial fibrillation	8744 (14.2)	1338 (17.7)	7406 (13.7)	<0.001
Coagulopathy	2699 (4.4)	485 (6.4)	2214 (4.1)	<0.001
Collagen vascular disease	2683 (4.4)	282 (3.7)	2401 (4.4)	0.117
Fluid/electrolyte disorders	18,576 (30.2)	2658 (35.2)	15,918 (29.5)	<0.001
Obesity	6554 (10.6)	537 (7.1)	6017 (11.1)	<0.001
Other neurological disorders	5196 (8.4)	599 (7.9)	4597 (8.5)	0.429
Pulmonary circulatory disease	1243 (2.0)	252 (3.3)	991 (1.8)	<0.001
Valvular heart disease	1592 (2.6)	296 (3.9)	1296 (2.4)	<0.001
Median household income				<0.001
First quartile	16,024 (26.4)	1739 (23.3)	14,285 (26.8)	
Second quartile	15,865 (26.1)	1957 (26.2)	13,908 (26.1)	
Third quartile	14,949 (24.6)	1816 (24.3)	13,133 (24.7)	
Fourth quartile	13,849 (22.8)	1953 (26.2)	11,896 (22.4)	
Primary payer				<0.001
Medicare	37,851 (61.5)	5462 (72.4)	32,389 (59.9)	
Medicaid	4028 (6.5)	389 (5.2)	3639 (6.7)	
Private including HMO	15,388 (25.0)	1435 (19.0)	13,953 (25.8)	
Self-pay/no charge/other	4315 (7.0)	256 (3.4)	4059 (7.5)	
Hospital characteristics				
Hospital teaching status				<0.001
Teaching	35,931 (58.3)	4799 (63.6)	31,132 (57.6)	
Nonteaching	25,652 (41.7)	2743 (36.4)	22,909 (42.4)	
Hospital location				0.149
Rural	212 (0.3)	13 (0.2)	199 (0.4)	
Urban	61,371 (99.7)	7529 (99.8)	53,842 (99.6)	
Hospital bed size				0.240
Small	4612 (7.5)	503 (6.7)	4109 (7.6)	
Medium	13,769 (22.4)	1643 (21.8)	12,126 (22.4)	
Large	43,203 (70.2)	5396 (71.5)	37,807 (70.0)	
Length of stay > 5 days	18,588 (30.2	3134 (41.6)	15,454(28.6)	<0.001
Disposition				<0.001
Home	44,092 (71.6)	4552 (60.4)	39,540 (73.2)	
Facility ^§^	15,710 (25.5)	2632 (34.9)	13,078 (24.2)	
AMA/unknown	1777 (2.9)	358 (4.7)	1419 (2.6)	
Mood disorders	11,502 (18.7)	11,354 (18.0)	10,148 (18.8)	0.378
Substance abuse	2676 (4.3)	165 (2.2)	2511 (4.6)	<0.001
Delirium/Dementia	2667 (4.3)	365 (4.8)	2302 (4.3)	0.213
Personality disorder	103 (0.2)	0 (0)	103 (0.2)	0.061
Acute decompensated heart failure	10,571 (17.2)	1472 (19.5)	9099 (16.8)	0.003
Cardiogenic shock	3564 (5.8)	582 (7.7)	2982 (5.5)	<0.001
Cardiac arrest	1663 (2.7)	221 (2.9)	1442 (2.7)	0.544
Postop stroke (complication)	792 (1.3)	103 (1.4)	689 (1.3)	0.704
Arrhythmia	16,868 (27.4)	2394 (31.7)	14,474 (26.8)	<0.001

Abbreviations: SE, standard error; PCI, percutaneous coronary intervention; CABG, coronary artery bypass grafting; HMO, health maintenance organization; AMA, against medical advice. * Values are presented as number (percentage) of patients unless otherwise indicated. † Survey-specific linear regression was performed. ‡ Rao-Scott χ^2^ test was used for all statistical tests unless stated otherwise. § Facility includes skilled nursing facility, intermediate care facility, and inpatient rehabilitation facility.

**Table 2:** In-hospital and 30-day Outcomes of Takotsubo Syndrome.

		Unadjusted		Adjusted *	
Outcomes	N (%)	OR (95% CI)	*p* Value	OR (95% CI)	*p* Value
Number of patients	7542 (12.2)				
Inhospital mortality					
Overall	1472 (2.4)				
No malignancy	1161 (2.1)	1 (Ref)		1 (Ref)	
Malignancy	311 (4.1)	1.96 (1.56–2.46)	<0.001	1.68 (1.29–2.17)	<0.001
30-day readmission mortality					
Overall	254 (0.4)				
No malignancy	205 (0.4)	1 (Ref)			
Malignancy	49 (0.6)	1.73 (0.88–3.42)	0.114	-	
30-day total mortality †					
Overall	1726 (2.8)				
No malignancy	1366 (2.5)	1 (Ref)		1 (Ref)	
Malignancy	360 (4.8)	1.96 (1.56–2.46)	<0.001	1.62 (1.25–2.10)	<0.001
30-day readmission					
Overall	7173 (11.6)				
No malignancy	5971 (11.0)	1 (Ref)		1 (Ref)	
Malignancy	1202 (15.9)	1.53 (1.34–1.75)	<0.001	1.33 (1.15–1.53)	<0.001

Abbreviations: OR, odds ratio; CI, confidence interval; Ref, reference. * Survey-specific multivariate generalized linear model was created with each outcome including all predictors with *p* values < 0.1 in the univariate analysis. Covariates for in-hospital mortality and 30-day total mortality are available in the Tables S1 and S2. † Thirty-day total mortality included in-hospital mortality and 30-day readmission mortality together.

**Table 3:** Independent Predictors of 30-Day Readmission After Index Hospitalization with Takotsubo Syndrome.

Variables	Unadjusted OR	Lower CI	Higher CI	*p* Value	Adjusted OR *	Lower CI	Higher CI	*p* Value
Malignancy	1.53	1.34	1.75	<0.001	1.33	1.15	1.53	<0.001
Length of stay > 5 days	2.45	2.23	2.69	<0.001	1.48	1.31	1.68	<0.001
Age ≥ 70 yrs	1.34	1.22	1.46	<0.001	0.95	0.84	1.07	0.378
Diabetes mellitus	1.27	1.13	1.42	<0.001	1.14	1.01	1.28	0.035
Family hx of CAD	0.56	0.46	0.69	<0.001	0.78	0.63	0.97	0.024
Chronic pulmonary disease	1.69	1.54	1.86	<0.001	1.40	1.27	1.55	<0.001
Chronic Kidney disease	1.88	1.64	2.16	<0.001	1.36	1.17	1.59	<0.001
Anemia	1.90	1.70	2.12	<0.001	1.26	1.12	1.42	<0.001
Atrial fibrillation	1.61	1.43	1.81	<0.001	1.24	1.08	1.41	0.002
Fluid and electrolyte disturbance	1.75	1.58	1.92	<0.001	1.20	1.07	1.34	0.001
Median household income (Ref: 1st quartile)								
Second quartile	0.92	0.80	1.04	0.188	0.97	0.85	1.11	0.689
Third quartile	0.83	0.72	0.94	0.004	0.90	0.79	1.03	0.10
Fourth quartile	0.75	0.66	0.85	<0.001	0.85	0.74	0.97	0.01
Primary payer (Ref: Medicare)								
Medicaid	1.11	0.93	1.31	0.250	1.25	1.02	1.53	0.032
Private including HMO	0.53	0.46	0.60	<0.001	0.75	0.64	0.88	<0.001
Self-pay/no charge/other	0.61	0.50	0.74	<0.001	0.82	0.66	1.00	0.054
Disposition (Ref: Home)								
Facility	2.60	2.35	2.87	<0.001	1.65	1.46	1.87	<0.001
AMA/unknown	0.37	0.22	0.62	<0.001	0.25	0.15	0.41	<0.001

Abbreviations: OR, odds ratio; CI, confidence interval; CAD, coronary artery disease; HMO, health maintenance organization; AMA, against medical advice. * A survey-specific multivariate generalized linear model was created with an outcome of 30-day readmission including all predictors with *p* values < 0.1 in the univariate analysis. Covariates for in hospital mortality and 30-day total mortality are available in the Tables S1 and S2.

**Table 4:** Independent Predictors of Higher 30-Day Total Cost of Hospitalization in Patients with Takotsubo syndrome.

	Unadjusted				Adjusted *			
Variables	Beta	Lower CI	Higher CI	*p* Value	Beta	Lower CI	Higher CI	*p* Value
Malignancy	0.093	0.078	0.108	<0.001	0.013	0.005	0.022	0.003
30-day readmission	0.327	0.312	0.341	<0.001	0.223	0.212	0.234	<0.001
Length of stay > 5 days	0.459	0.449	0.469	<0.001	0.31	0.301	0.318	<0.001
Age ≥ 70 yrs	0.033	0.023	0.042	<0.001	−0.026	−0.033	−0.018	<0.001
Female (Ref: male)	−0.109	−0.130	−0.089	<0.001	−0.048	−0.061	−0.036	<0.001
Diabetes mellitus	0.041	0.029	0.052	<0.001	0.015	0.007	0.022	<0.001
Dyslipidemia	−0.070	−0.080	−0.061	<0.001	−0.014	−0.02	−0.008	<0.001
Previous PCI	−0.024	−0.043	−0.005	0.015	−0.02	−0.034	−0.006	0.005
Family history of CAD	−0.125	−0.138	−0.112	<0.001	−0.013	−0.024	−0.003	0.009
Congestive heart failure	0.184	0.173	0.194	<0.001	0.027	0.019	0.034	<0.001
Peripheral vascular disease	0.121	0.102	0..139	<0.001	0.028	0.016	0.04	<0.001
Chronic pulmonary disease	0.107	0.096	0.118	<0.001	0.019	0.012	0.026	<0.001
Chronic kidney disease	0.169	0.150	0.188	<0.001	0.03	0.017	0.044	<0.001
Chronic liver disease	0.200	0.157	0.242	<0.001	0.04	0.012	0.068	0.005
Anemia	0.217	0.203	0.231	<0.001	0.031	0.022	0.041	<0.001
Atrial fibrillation	0.140	0.124	0.156	<0.001	0.018	0.008	0.028	<0.001
Coagulopathy	0.339	0.309	0.368	<0.001	0.097	0.078	0.115	<0.001
Drug abuse	0.114	0.089	0.140	<0.001	0.027	0.01	0.043	0.001
Fluid/electrolyte disorders	0.257	0.246	0.269	<0.001	0.059	0.051	0.068	<0.001
Other neurological disorders	0.132	0.112	0.152	<0.001	0.027	0.014	0.04	<0.001
Pulmonary circulatory disease	0.407	0.369	0.444	<0.001	0.096	0.065	0.127	<0.001
Valvular heart disease	0.323	0.293	0.353	<0.001	0.06	0.038	0.083	<0.001
Cardiogenic shock	0.418	0.396	0.440	<0.001	0.122	0.102	0.142	<0.001
Cardiac arrest	0.403	0.372	0.434	<0.001	0.147	0.122	0.173	<0.001
Intraaortic balloon pump	0.415	0.383	0.448	<0.001	0.086	0.058	0.114	<0.001
Median household income (Ref: first quartile)								
Second quartile	0.007	−0.008	0.023	0.339	0.027	0.018	0.036	<0.001
Third quartile	0.025	0.010	0.040	0.001	0.051	0.042	0.061	<0.001
Fourth quartile	0.037	0.022	0.053	<0.001	0.077	0.067	0.088	<0.001
Primary payer (Ref: medicare)								
Medicaid	0.041	0.020	0.062	<0.001	0.033	0.019	0.047	<0.001
Private	−0.083	−0.094	−0.072	<0.001	−0.003	−0.011	0.006	0.506
Self-pay/no charge/others	−0.069	−0.087	−0.050	<0.001	−0.001	−0.014	0.012	0.897
Disposition (Ref: home)								
Facility	0.316	0.304	0.327	<0.001	0.077	0.069	0.086	<0.001
AMA/unknown	0.346	0.311	0.380	<0.001	0.116	0.091	0.141	<0.001
Year (per year)	0.009	0.004	0.014	<0.001	0.006	−0.000	0.011	0.060

Abbreviations: TTS, Takotsubo syndrome; PCI, percutaneous coronary intervention; CAD, coronary artery disease. * A survey-specific multivariate linear regression model was created with an outcome of log-transformed cumulative cost including all predictors with *p* values < 0.1 in the univariate analysis. Hospital ID was also included as a covariable for consideration of hospital fixed-year effect (insignificant contribution, not shown).

## Data Availability

The data presented in this study are available on request from the corresponding author. Original United States Nationwide Readmissions Database used for this study is available at https://www.hcup-us.ahrq.gov/db/nation/nrd/nrddbdocumentation.jsp (accessed on 1 May 2021).

## Supplementary Materials

The following are available online at https://www.mdpi.com/article/10.3390/jcm10163701/s1, Figure S1: Cumulative Density Function for Readmissions within 30 Days in Takotsubo syndrome, Table S1: Independent Predictors of In-hospital Mortality After Index Hospitalization With Takotsubo Syndrome, Table S2: Independent Predictors of 30-Day Total Mortality After Index Hospitalization With Takotsubo Syndrome, Table S3: Causes of Readmission in all Takotsubo Syndrome Patients, Table S4: Causes of Readmission in Takotsubo Syndrome Patients With Malignancy, Table S5: Causes of Readmission in Takotsubo Syndrome Patients Without Malignancy, Table S6: Costs and Charges over 30 Days After Index Hospitalization for Takotsubo syndrome With or Without Malignancy.
