# Supplementary material for: Thirty-Day Readmission Rates after Takotsubo Syndrome with or without Malignancy: A Nationwide Readmissions Database Analysis
Source: J Clin Med. 2021 Aug 20;10(16):3701. doi: 10.3390/jcm10163701 (PMC8397058; doi:10.3390/jcm10163701)
Supplement: Supplementary file 1 [file jcm-10-03701-s001.zip › jcm-1343088-supplementary.pdf]

Online Supplementary Materials

Figure S1. Cumulative Density Function for Readmissions within 30 Days in Takotsubo syndrome

Table S1. Independent Predictors of In-hospital Mortality After Index Hospitalization With Takotsubo Syndrome

Table S2. Independent Predictors of 30-Day Total Mortality After Index Hospitalization With Takotsubo Syndrome

Table S3. Causes of Readmission in all Takotsubo Syndrome Patients

Table S4. Causes of Readmission in Takotsubo Syndrome Patients With Malignancy

Table S5. Causes of Readmission in Takotsubo Syndrome Patients Without Malignancy

Table S6. Costs and Charges over 30 Days After Index Hospitalization for Takotsubo syndrome With or Without Malignancy

Figure S1. Cumulative Density Function for Readmissions within 30 Days in Takotsubo syndrome

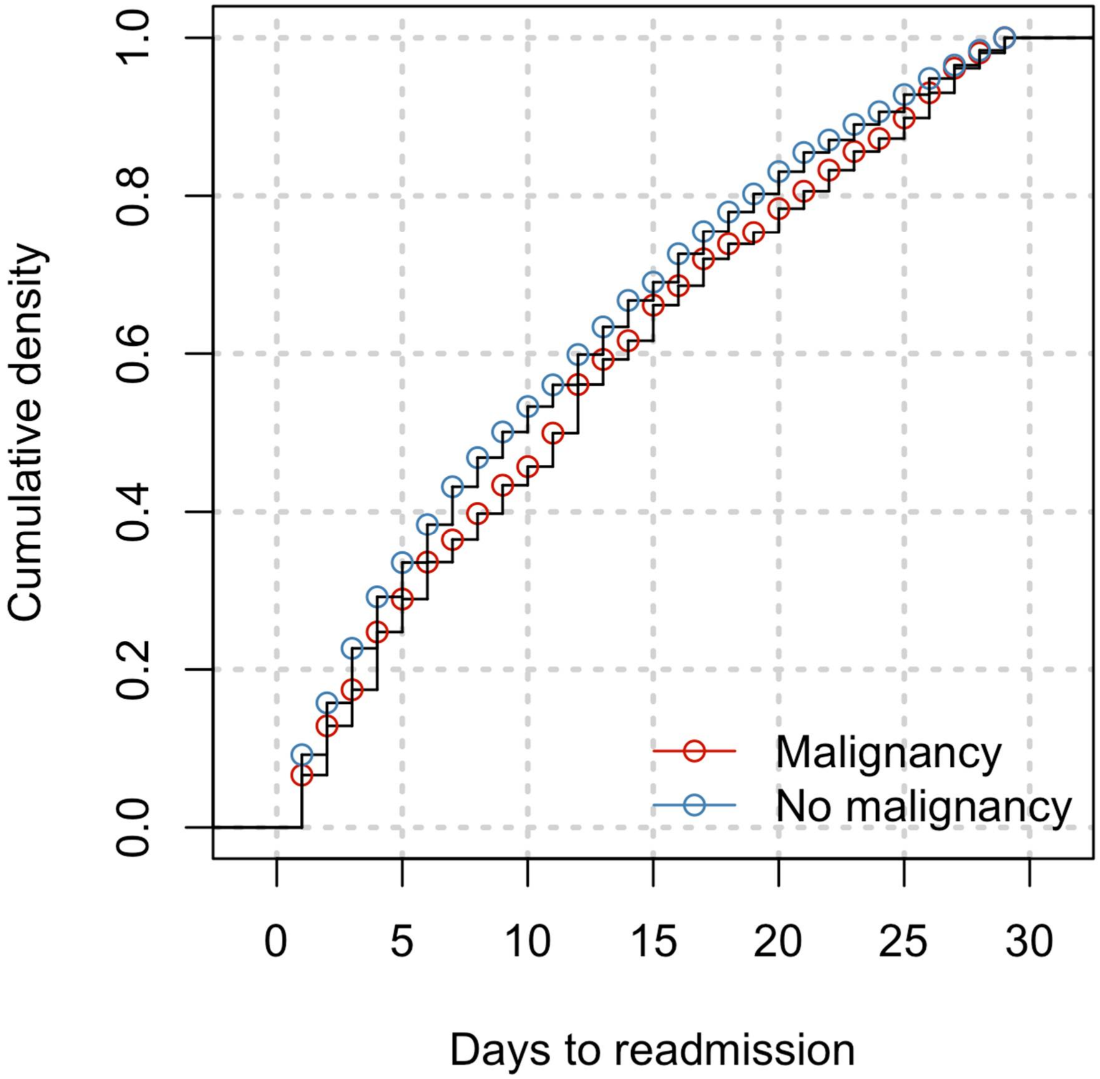

Table S1. Independent Predictors of In-hospital Mortality After Index Hospitalization With Takotsubo Syndrome

Variables	Unadjusted OR	Lower CI	Higher CI	P value	Adjusted OR	Lower CI	Higher CI	P value
Malignancy	1.96	1.56	2.46	<0.001	1.68	1.29	2.17	<0.001
Age≥70 yrs	1.81	1.49	2.19	<0.001	1.85	1.49	2.30	<0.001
Smoking	0.71	0.58	0.88	<0.001	0.72	0.57	0.91	0.006
Fluid and electrolyte	3.80	3.17	4.56	<0.001	2.06	1.67	2.55	<0.01
Pulmonary Circ Disease	3.81	2.65	5.48	<0.001	1.86	1.18	2.93	0.008
Chronic Pulmonary disease	1.41	1.15	1.74	<0.001	1.31	1.04	1.65	0.022
Cardiogenic shock	11.26	9.18	13.82	<0.001	5.80	4.53	7.43	<0.001
Cardiac arrest	16.88	13.10	21.74	<0.001	8.63	6.34	11.76	<0.001
Arrhythmia	2.66	2.19	3.24	<0.001	1.62	1.31	2.01	<0.001

Abbreviations: OR, odds ratio; CI, confidence interval.

Table S2. Independent Predictors of 30-Day Total Mortality After Index Hospitalization With Takotsubo Syndrome*

Variables	Unadjusted OR	Lower CI	Higher CI	P value	Adjusted OR	Lower CI	Higher CI	P value
Malignancy	1.93	1.55	2.42	<0.001	1.62	1.25	2.10	<0.001
Age≥70 yrs	1.87	1.55	2.26	<0.001	1.88	1.51	2.34	<0.001
Smoking	0.75	0.63	0.93	<0.001	0.77	0.62	0.96	0.019
Fluid and electrolyte	3.74	3.14	4.45	<0.001	2.15	1.76	2.63	<0.001
Pulmonary Circ Disease	3.85	2.68	5.55	<0.001	1.90	1.22	2.96	<0.001
Chronic pulmonary disease	1.58	1.31	1.90	<0.001	1.45	1.18	1.79	<0.001
Cardiogenic shock	9.39	7.66	11.51	<0.001	4.82	3.78	6.16	<0.001
Cardiac arrest	14.18	11.08	18.16	<0.001	7.60	5.64	10.26	<0.001
Arrhythmia	2.47	2.05	2.97	<0.001	1.52	1.24	1.85	<0.001
Family Hx of CAD	0.36	0.21	0.63	<0.001	0.58	0.34	0.98	0.042

Abbreviations: OR, odds ratio; CI, confidence interval; CAD, coronary artery disease.

*Thirty-day total mortality included inhospital mortality and 30-day readmission mortality together.

Table S3. Causes of Readmission in all takotsubo syndrome patients

Causes of Readmission	CCS codes	Frequency (%)
**Cardiac causes**		**21.6**
Heart failure	108	10.62
Arrhythmia	106	4.70
Angina and Chronic Ischemic Heart Disease	101	2.60
Acute myocardial infarction	100	2.23
Non-specific chest pain	102	1.93
Cardiac arrest	107	1.10
**Other cardiac causes**		**9.0**
Other circulatory (heart) disease/Complication of hypertension	98, 99, 104, 117	2.83
Syncope	245	0.73
Myocarditis/pericarditis	97	0.70
Valvular heart disease	96	0.45
Conduction disorder	105	0.40
**Non-cardiac causes**		**69.4**
Infectious	2, 4, 122, 135, 148, 159, 197, 201	19.76
Respiratory	103, 127, 128, 129*, 130, 131, 133, 134	12.44
Others	81, 85, 199, 205, 211, 212, 231, 248, 251, 252, 259, N/S	6.9
Gastrointestinal	120, 138, 140, 141, 145, 146, 149, 151, 152, 153, 154, 155, 230, 246, 250	6.39
Endocrine/Metabolic	50, 51, 55, 58	4.4
Neuropsychiatric/Substance	83, 95, 242, 653, 657, 659, 660, 661	3.42
Genitourinary	157, 158, 163, 164	3.08
Hematologic/oncologic	19, 42, 45, 59, 60, 62	1.99
Stroke/Transient ischemic attack	109, 110, 112, 113	1.56
Peripheral vascular disease	114, 115, 116, 118	1.48
Trauma	226, 233, 234, 244	1
Complication of medical procedure or implant/graft	237, 238	8.8
Rehabilitation	254	1.14

*Aspiration pneumonitis (CCS 129) was included in the respiratory problem.

Table S4. Causes of Readmission in takotsubo syndrome patients with malignancy

Causes of Readmission	CCS codes	Frequency (%)
**Cardiac causes**		**18.3**
Heart failure	108	8.1
Arrhythmia	106	4.9
Angina and Chronic Ischemic Heart Disease	101	1.8
Acute myocardial infarction	100	0.8
Non-specific chest pain	102	2.6
Cardiac arrest	107	0.1
**Other cardiac causes**		**6.2**
Other circulatory (heart) disease/Complication of hypertension	98, 99, 104, 117	4.3
Syncope	245	0.2
Myocarditis/pericarditis	97	1.6
Valvular heart disease	96	0.1
Conduction disorder	105	0
**Non-cardiac causes**		**75.5**
Infectious	2, 4, 122, 135, 148, 159, 197, 201	20.1
Respiratory	103, 127, 128, 129*, 130, 131, 133, 134	8.4
Others	81, 85, 199, 205, 211, 212, 231, 248, 251, 252, 259, N/S	10.9
Gastrointestinal	120, 138, 140, 141, 145, 146, 149, 151, 152, 153, 154, 155, 230, 246, 250	7.8
Endocrine/Metabolic	50, 51, 55, 58	3.1
Neuropsychiatric/Substance	83, 95, 242, 653, 657, 659, 660, 661	3.8
Genitourinary	157, 158, 163, 164	4.6
Hematologic/oncologic	19, 42, 45, 59, 60, 62	6.7
Stroke/Transient ischemic attack	109, 110, 112, 113	2.5
Peripheral vascular disease	114, 115, 116, 118	1.2
Trauma	226, 233, 234, 244	0.9
Complication of medical procedure or implant/graft	237, 238	5.5
Rehabilitation	254	0

*Aspiration pneumonitis (CCS 129) was included in the respiratory problem.

Table S5. Causes of Readmission in takotsubo syndrome patients without malignancy

Causes of Readmission	CCS codes	Frequency (%)
**Cardiac causes**		**22.3**
Heart failure	108	11.14
Arrhythmia	106	3.29
Angina and Chronic Ischemic Heart Disease	101	2.42
Acute myocardial infarction	100	1.67
Non-specific chest pain	102	3.5
Cardiac arrest	107	0.28
**Other cardiac causes**		**9.5**
Other circulatory (heart) disease/Complication of hypertension	98, 99, 104, 117	6.46
Syncope	245	0.87
Myocarditis/pericarditis	97	1.55
Valvular heart disease	96	0.35
Conduction disorder	105	0.27
**Non-cardiac causes**		**68.1**
Infectious	2, 4, 122, 135, 148, 159, 197, 201	12.04
Respiratory	103, 127, 128, 129*, 130, 131, 133, 134	12.41
Others	81, 85, 199, 205, 211, 212, 231, 248, 251, 252, 259, N/S	8.8
Gastrointestinal	120, 138, 140, 141, 145, 146, 149, 151, 152, 153, 154, 155, 230, 246, 250	9.45
Endocrine/Metabolic	50, 51, 55, 58	3.7
Neuropsychiatric/Substance	83, 95, 242, 653, 657, 659, 660, 661	4.75
Genitourinary	157, 158, 163, 164	3.62
Hematologic/oncologic	19, 42, 45, 59, 60, 62	0.99
Stroke/Transient ischemic attack	109, 110, 112, 113	3.96
Peripheral vascular disease	114, 115, 116, 118	2.33
Trauma	226, 233, 234, 244	1.2
Complication of medical procedure or implant/graft	237, 238	4.85
Rehabilitation	254	0.11

*Aspiration pneumonitis (CCS 129) was included in the respiratory problem.

Tale S6. Costs and Charges over 30-days after Index Hospitalization for Takotsubo syndrome with or without Malignancy

Outcomes	Overall	TTS with malignancy	TTS without malignancy	P Value†
**Number of patients, n (%)**	61583 (100)	7542 (12.2)	54041 (87.8)	
**Charge (index hospitalization), $**	39498 (25935-68521)*	46876 (28416-87536	38593 (25481-66425)	<0.001
**Cumulative charges (index hospitalization + 30-day readmission), $**	41830 (26501-75167)	50936 (29819-97989)	40735 (26160-72619)	<0.001
**Cost (index hospitalization), $**	11427 (7999-17933)	13344 (8909-24171)	11194 (7900-17933)	<0.001
**Cumulative costs (index hospitalization + 30-day readmission), $**	11998 (8181-20564)	14686 (9294-27337)	11704 (8065-19630)	<0.001

Abbreviations: TTS, Takotsubo syndrome.

*All values are shown as a median (interquartile range).

†Mann-Whitney-Wilcoxon test was used in all comparisons.
